# Olfactory Learning Behavior and Mortality of the Honey Bee *Apis mellifera jemenitica* in Response to Pyrethroid Insecticide (Deltamethrin)

**DOI:** 10.3390/toxics12010025

**Published:** 2023-12-28

**Authors:** Mohamedazim I. B. Abuagla, Javaid Iqbal, Hael S. A. Raweh, Abdulaziz S. Alqarni

**Affiliations:** Department of Plant Protection, College of Food and Agriculture Sciences, King Saud University, P.O. Box 2460, Riyadh 11451, Saudi Arabiajiqbal@ksu.edu.sa (J.I.);

**Keywords:** honey bee, Saudi Arabia: toxicity, insecticide, lethal concentration, learning, memory

## Abstract

Honey bees are constantly threatened due to the wide use of pesticides. This study presents the effects of deltamethrin on the mortality, olfactory learning, and memory formation of the native Saudi bee *Apis mellifera jemenitica*. Topical and oral application of realistic field and serial dilutions of deltamethrin (250, 125, 62.5, and 25 ppm) caused significant mortality at 4, 12, 24, and 48 h posttreatment. Bee mortality increased with the increasing concentration of insecticide at all tested posttreatment times. Highest mortality was observed at 24 h and 48 h after both exposure routes. Food consumption gradually decreased with increasing concentration of deltamethrin during oral exposure. The LC_50_ of deltamethrin was determined at 12, 24, and 48 h for topical (86.28 ppm, 36.16 ppm, and 29.19 ppm, respectively) and oral (35.77 ppm, 32.53 ppm, and 30.78 ppm, respectively) exposure. Oral exposure led to significantly higher bee mortality than topical exposure of deltamethrin at 4 h and 12 h, but both exposure routes were equally toxic to bees at 24 h and 48 h. The sublethal concentrations (LC_10_, LC_20_, and LC_30_) of deltamethrin significantly impaired the learning during conditioning trials, as well as the memory formation of bees at 2, 12, and 24 h after topical and oral exposure. Thus, deltamethrin inhibits learning, and bees were unable to memorize the learned task.

## 1. Introduction

Honey bees pollinate approximately 70% of major crop species [1,2,3,4]. Bee pollination enhances both the quality and output of crops [5]. During the flowering season, bees visit flowers in search of nectar and pollen, producing honey and other useful products such as propolis, royal jelly, bee pollen, bee bread, bee venom, and wax [6]. Bees are also crucial for maintaining ecosystem balance and biodiversity [7].

The sudden disappearance and death of a large number of honey bees from their hives in recent years is a serious and alarming issue that adversely affects beekeeping, pollination, and food production in general [8]. Multiple biotic and abiotic factors—including diseases, *Varroa* infestation, environmental degradation, beekeeping practices, and agrochemicals—are under investigation in relation to colony collapse disorder (CCD) in honey bees [8,9,10,11]. Insecticidal exposure is the main cause of the decline in honeybee populations [12,13,14,15,16,17].

Bees are exposed to insecticides in different ways, such as by direct spraying during foraging (topical/contact exposure) or during flower visitations (contact/oral exposure) [18,19]. The toxicity effects of pesticides differ due to their chemical nature, method of exposure, and whether the exposure concentration is lethal or sublethal [17,20]. Both lethal and sublethal concentrations of insecticides are harmful and cause numerous acute or chronic toxic impacts on bee hives, including reductions in reproduction, immunity, foraging, homing, lifespan, colony health, cognition, and overall physiological functioning; behavioral changes; and many more [12,21,22,23,24,25,26]. Insecticidal exposure is not confined only to crops; weeds, bushes, and ornamental plants in the vicinity can also lead to the poisoning of forager bees [27]. Additionally, bees can also be exposed to insecticides in multiple other ways, such as poisoning through nearby contaminated water of streams/ponds, spraying in nearby areas drifting towards bee colonies, and the collection of contaminated nectar and pollens [12,28].

In Saudi Arabia, pesticide consumption has increased rapidly in the last three decades due to expansion of agricultural areas and an increase in food demand [29]. The broad use of pesticides against a variety of agricultural and domestic insect pests is becoming an alarming risk to the environment, human health, and pollinators, especially honey bees [30,31,32,33,34]. Pyrethroids—a group of insecticides—have extreme effects on honey bees, especially in agricultural areas [35], including frequent detection of their residues in honey bee products [36]. Pyrethroids are also frequently used against health-oriented insects and other agricultural insects in Saudi Arabia [15,37,38]. Deltamethrin is a broad-spectrum pyrethroid insecticide widely used against mosquitoes, locusts, and other agricultural pests worldwide, including Saudi Arabia [37,38,39,40,41]. Deltamethrin affects insects when they ingest it or come in direct contact with it [23,41]. It is a neurotoxic insecticide that imposes numerous harmful effects against bees, such as a reduction in fecundity; a prolonged immature stage; and disruption in larval development, homing flight ability, orientation, dance communications, lifespan, biology, and foraging activity [17,24,42,43,44,45].

Honey bees have strong olfactory senses through their antennae and proboscis, which help them during foraging, mating, and social communications [46]. Honey bees can discriminate flowers and nectar through olfactory behavior [47]. Olfactory behavior is important for bees inside or outside of hives to find a good source of food, navigation and discrimination of nestmates from alien individuals [48]. Olfactory sensation, a major contributor to foraging behavior, is also vulnerable to different factors, including pathogens [49], air pollution [50] and insecticides [51]. The higher functionality of the olfactory sensory system is affected by exposure to even small amounts of pesticide [12]. Therefore, any impact on the olfaction and brains of bees by lethal and sublethal concentrations of insecticides may lead to disruption in cognitive capabilities [52], and bees can become incapable of locating food sources [53].

Beekeeping in Saudi Arabia plays a vital role in supporting rural communities by providing economic opportunities, promoting biodiversity, sustaining the environment, and enhancing crop production through pollination [54,55]. The native bee in Saudi Arabia, *Apis mellifera jemenitica* (Ruttner, 1976), is uniquely adapted to hot and arid environments [56]. It has been used in apiculture since before 2000 BC in the Arabian Peninsula, indicating its enduring importance and effectiveness due to its ability to survive in extreme temperatures and its foraging capabilities, which make it highly suited for the conditions in western and central Saudi Arabia [57,58,59].

Considering the potential poisoning of bees, it is alarming that native bees (*A. m. jemenitica*) are at potential massive risk due to insecticides being used against agricultural and public health insect pests. Therefore, in this study, the effects of deltamethrin via two exposure routes (topical and oral) on the mortality, learning, and memory formation of honey bee foragers *A. m. jemenitica* was evaluated under laboratory conditions.

## 2. Materials and Methods

### 2.1. Capture and Conservation of Bees

**Figure 1:**
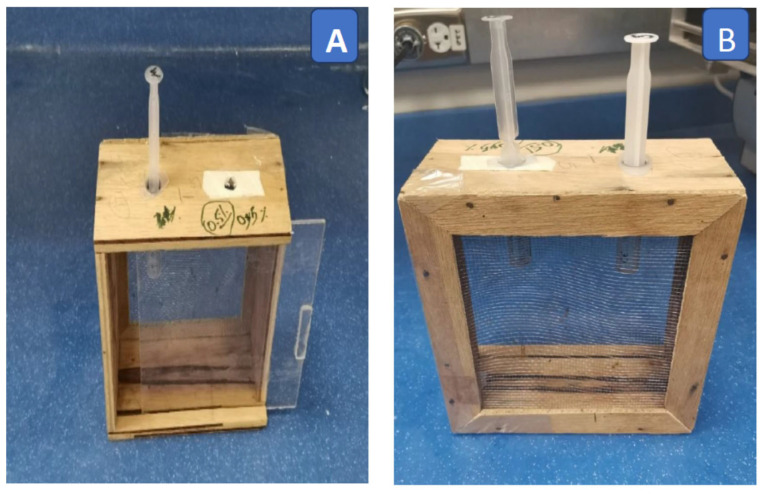
Wooden cage (15 × 15 × 5 cm: L × H × W). (**A**) Front side with Plexiglass; (**B**) back side of cage with mesh.

Colonies of *A. m. jemenitica* were reared at the educational farm (24°44′14.2″ N 46°37′09.9″ E) of the Food and Agriculture Sciences College on the campus of King Saud University, Riyadh, Saudi Arabia. The source colonies of *A. m. jemenitica* were acquired from the Ministry of Environment, Water and Agriculture, Kingdom of Saudi Arabia. The hives were kept healthy, without any insecticide treatment, and free of any infectious pathogens (*Nosema* sp. and parasitic *Varroa* mites). The adult forager bees (80–100) were caught randomly from the hive entrances, and bees were maintained in wooden cages (15 × 15 × 5 cm: L × H × W) with Plexiglass on the front side and wire mesh on the back (Figure 1). The cages with the bees were placed in an incubator (LIB-060M, Lab Tech, Daihan Lab Tech Co., Ltd., Namyangju- City, Gyeonggi–Do, Korea) for adaptation inside the cages at 25 ± 2 °C and 60 ± 10% relative humidity (R.H.) for two hours before the subsequent bioassay [60,61].

### 2.2. Insecticides and Application

The commercial formulation of the insecticide deltamethrin (Klash^®^ 25 EC, 25 g/L, Astrachem, Astra Industrial Complex Co., Ltd., Dammam, Saudi Arabia) was purchased from a local market in Riyadh, Saudi Arabia. Serial dilutions (250, 125, 62.5, and 25 ppm) were prepared from the recommended field rate (250 ppm) of this formulation against mosquitos, locusts, cockroaches, flies, bedbugs, termites, and many other insects. Two exposure routes of insecticide (topical application and oral feeding) were applied to determine bee mortality. The mortality data in response to all tested dilutions were used to calculate the lethal concentration (LC_50_). Acetone for topical application and a sugar solution for oral feeding were used as control treatments in the respective experiments.

### 2.3. Toxicity Bioassay

#### 2.3.1. Topical Application

**Figure 2:**
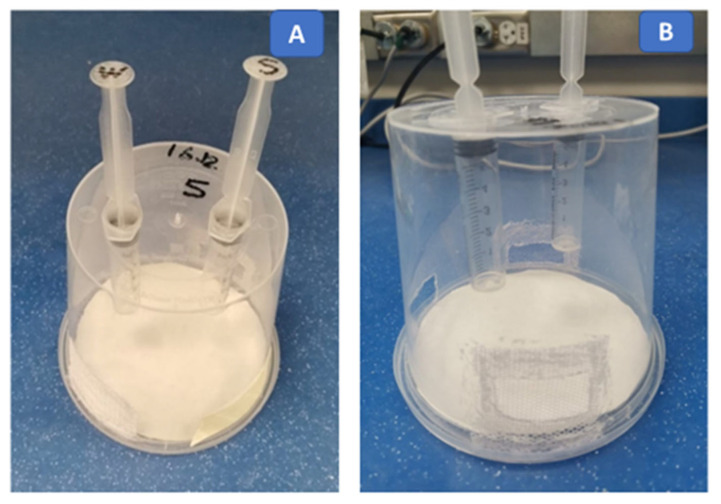
Plastic containers for the toxicity bioassay. (**A**) Syringes—W for water, S for sugar; (**B**) meshes installed on each side of container for proper ventilation.

Serial test dilutions (250, 125, 62.5, and 25 ppm) of deltamethrin were prepared in acetone (organic solvent). Acetone alone was applied on the thorax as a control treatment [62]. Ten bees were taken randomly from the wooden cage (Figure 1) and immobilized on ice for 3–5 min. Using a micropipette, 1 μL of each serial test concentration of deltamethrin was applied topically on the dorsal side of the thorax [63,64]. Ten treated bees were released in each plastic container [65]. The treated bees were provided with a 50% (*w*/*v*) sucrose solution and water in the plastic container using separate plastic syringes (5 mL) as feeder units (Figure 2) [66,67]. The plastic containers with treated bees were kept in an incubator at 25 ± 2 °C and 60 ± 10% RH. The number of dead bees was recorded at 4, 12, 24, and 48 h and after treatment to estimate the mortality of bees exposed to different insecticide dilutions. If the bees did not move after being touched by a fine brush, they were considered dead [66]. The experiment was repeated four times [68] with each serial dilution of deltamethrin.

#### 2.3.2. Oral Feeding

The forager bees in the wooden cages were starved for 2 h in an incubator (25 ± 2 °C and 60 ± 10% RH) before oral treatment to facilitate subsequent feeding. Ten bees were randomly taken from the wooden cage (Figure 1) and transferred to plastic containers (Figure 2) for each test dilution [65]. Serial test dilutions (250, 125, 62.5, and 25 ppm) of deltamethrin were prepared in a 50% sucrose solution (*w*/*v*). Two hundred microliters of sucrose solution with insecticides of each test dilution was given in each plastic container. The plastic containers were kept in the dark at 25 ± 2 °C and 60 ± 10% RH, and the bees consumed food for 4 h [65,69]. The sucrose solution alone was used in separate plastic containers as a control treatment [65]. The quantity of consumed food per group after 4 h of feeding was monitored. The percentage of food consumption was calculated after the administration of each test dilution of deltamethrin. To ensure the consumption of all ingested food by the bees, the treated bees were kept for an hour without any food. The bees were then provided 50% sucrose solution (*w*/*v*) alone and water [65] ad libitum to satisfy their food requirements for survival. The sucrose solution and water were replaced daily until the completion of the experiment. The number of dead bees was counted at 4, 12, 24, and 48 h after feeding, and the mortality of bees was calculated. The experiment was repeated four times [68,70] with serial dilutions of deltamethrin.

### 2.4. Probit Analysis

Mortality (%) was calculated for each concentration at different time periods (4 h, 12 h, 24 h, and 48 h). The Abbott formula was applied for the corrected mortality of individuals who responded during each treatment [71].

$$\mathrm{Corrected}\;\mathrm{Mortality}=\left(\frac{\mathrm{Mort}\%\;\mathrm{in}\;\mathrm{Tr}\;\mathrm{plot}-\mathrm{Mort}\;\%\;\mathrm{in}\;\mathrm{Cont}\;\mathrm{plot}}{100-\mathrm{Mort}\;\%\;\mathrm{in}\;\mathrm{Cont}\;\mathrm{plot}}\right)\times 100$$

The lethal concentrations were then obtained, together with 95% confidence upper and lower limits, using probit analysis according to [72] with LdP Line software [73].

### 2.5. Olfactory Learning Trials and Memory Tests

The adult forager bees were caught randomly from the hive entrance and placed directly in small plastic tubes after immobilization on ice for 3–5 min in the laboratory [58,74]. Each bee’s head was fixed with dental wax to avoid free movement. The bees were fed 0.5 M sucrose and kept overnight in a dark, moist place at 25 ± 2.0 °C and 50 ± 10% RH conducive to their survival.

Honey bees were exposed to 1 µL of three sublethal concentrations (LC_10_, LC_20_, and LC_30_) of deltamethrin at 24 h as topical application and oral feeding 1 h prior to conditioning trials [22]. The bees were initially motivated with 0.5 M sucrose solution 10 min prior to the actual learning trials by touching the antenna without feeding. The bees responding to the initial motivation were used in subsequent learning experiments [58,74,75]. The bees were trained in three successive learning trials at 10 min intra-trial intervals following the protocol of proboscis extension response (PER) for associative learning: a conditioning trial that comprises pairing odor stimulus (CS-conditioned stimulus: odor of clove oil) with an appetitive reward stimulus (US-unconditioned stimulus: 1 M sucrose) [58,74,76]. Memory formation was tested at 2, 12, and 24 h after conditioning using odor stimulus only. The PER of bees was recorded during conditioning trials and the memory formation tests. A conditioned PER was recorded as positive if the bee fully extended its proboscis during presentations of conditioned odor stimulus, and negative if the bee did not extend its proboscis [74,77]. The harnessed bees were fed 0.5 M sucrose solution to maintain the dietary requirements of the bees [22,58] during the experimental duration. The percentage of bees exhibiting PER to the conditioned odorant was calculated to present the level of learning and memory of the bees. Each sublethal concentration of deltamethrin was tested four times, with twenty bees in each replication for a total of eighty bees per concentration.

### 2.6. Statistical Analysis

The data on bee mortality after topical or oral exposure to deltamethrin at different time periods were subjected to probit analysis [72] to determine the lethal and sublethal concentrations of the insecticide. The corrected mortality was calculated using Abbott’s formula. The lethal (LC_50_/LC_90_) and sublethal concentrations (LC_10_, LC_20_, and LC_30_) and the regression equation were determined using LdP Line software [73]. The mortality data and food consumption during oral feeding were analyzed statistically using one-way ANOVA (SAS 9.2 software), followed by Duncan’s multiple range test for comparison of means. The proboscis extension response (PER) data from the honey bee learning and memory tests were analyzed using Pearson’s nonparametric Fisher’s exact test, or the chi-square (χ^2^) test (*p* < 0.05).

## 3. Results

### 3.1. Percent Mortality of Honey Bees

#### 3.1.1. Topical Application of Deltamethrin

**Figure 3:**
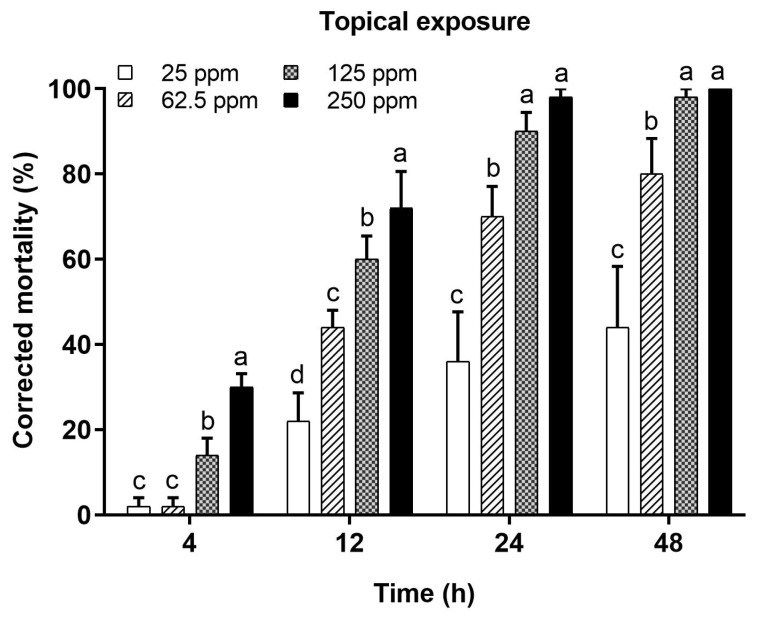
Mortality (%) of *Apis mellifera jemenitica* after topical application of deltamethrin. Different letters indicate significant differences among the tested concentrations at each timeframe.

The percent mortality was significantly different at the tested timeframes (4, 12, 24, and 48 h) after the topical application of 1 µL of deltamethrin on the thoraxes of honey bees at its realistic field concentration (250 ppm) and subsequent serial concentrations (125, 62.5, and 25 ppm) (Figure 3). The mortality increased with an increase in the concentration of insecticide at all tested posttreatment times. High mortality was recorded for topical applications of a higher concentration, and this mortality gradually decreased with a low concentration of deltamethrin. The mortality was significantly different among the tested concentrations at 4 h (F = 25.65; *p =* 0.0001), 12 h (F = 25.63; *p <* 0.0001), 24 h (F = 39.36; *p <* 0.0001), and 48 h (F = 32.16; *p <* 0.0001). The lowest mortality (below 30%) was recorded at 4 h posttreatment in all tested concentrations compared to the later posttreatment times (12 h, 24 h, and 48 h). Two high concentrations (250 ppm and 125 ppm) were significantly toxic at 12 h (72% and 60%, respectively), at 24 h (98% and 90%, respectively) and 48 h (100% and 98%, respectively). Furthermore, the mortality rates corresponding to two other concentrations (62.5 ppm and 25 ppm) were also significantly different at 12 h (44% and 22%, respectively), at 24 h (70% and 36%, respectively), and at 48 h (80% and 44%, respectively). In general, the mortality of honey bees during the investigated timeframes, specifically 12 h, 24 h, and 48 h, varied significantly among concentrations.

#### 3.1.2. Oral Application of Deltamethrin

**Figure 4:**
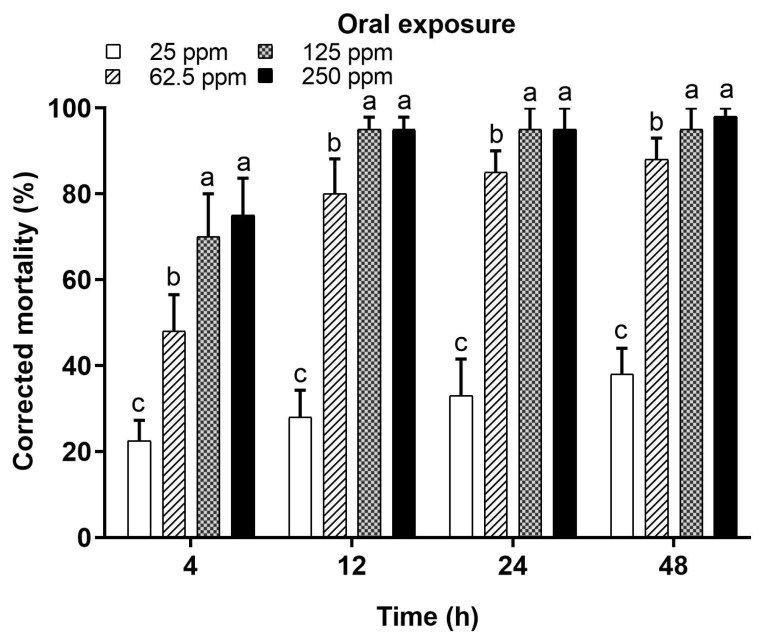
Mortality (%) of *Apis mellifera jemenitica* after oral administration of deltamethrin. Different letters indicate significant differences among the tested concentrations at each timeframe.

Serial dilutions of deltamethrin (250, 125, 62.5, and 25 ppm) were administered orally in a 50% sucrose solution. There were significant differences among the tested concentrations for the recorded mortality of honey bees. The mortality increased with an increase in the concentration of insecticide at all tested posttreatment times. High mortality was recorded with higher concentrations, which gradually decreased at low concentrations (Figure 4). The investigated concentrations resulted in significant differences in mortality at 4 h (F = 17.41; *p* < 0.0001), 12 h (F = 76.32; *p* < 0.0001), 24 h (F = 62.70; *p* < 0.0001), and 48 h (F = 99.10; *p* < 0.0001). The lowest mortality (22.5%, 28.0%, 33.0%, and 38.0%) was recorded only with the lowest tested concentration (25 ppm) at all tested timeframes (4, 12, 24, and 48 h, respectively). In comparison to topical exposure, oral intake of deltamethrin was more quickly toxic to honey bees. At 4 h, the mortality was 48%, 70%, and 75% at concentrations of 62.5 ppm, 125 ppm, and 250 ppm, respectively. The oral intake of deltamethrin resulted in greater than 50% mortality at 12 h, 24 h, and 48 h with all tested concentrations except the lowest one. Conclusively, the mortality of honey bees throughout the investigated timeframes (4, 12, 24, and 48 h) differed significantly between concentrations.

#### 3.1.3. Food Consumption during Oral Feeding (Sensitivity Response)

**Figure 5:**
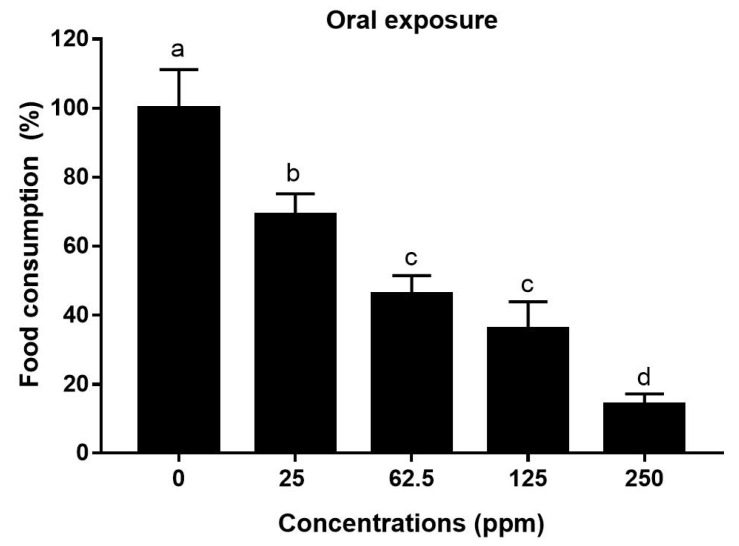
Food consumption (%) of *Apis mellifera jemenitica* during oral intake of deltamethrin. Different letters indicate significant differences among the tested concentrations.

During oral intake of deltamethrin, bees feeding on different tested concentrations revealed significantly different (F = 13.01; *p* < 0.0001) food consumption percentages. The untreated control group consumed all of the provided food (100%). The intake gradually decreased with an increase in the concentration of deltamethrin added to the food. Food consumption was 69, 46, and 36% after the administration of 25, 62.5, and 125 ppm deltamethrin, respectively. The lowest food consumption (14%) was observed with the highest concentration of deltamethrin (250 ppm) (Figure 5). Thus, oral exposure to high concentrations of deltamethrin led to decreased intake, but this intake was still enough to cause a lethal impact on the honey bees, as shown in Figure 5.

#### 3.1.4. Lethal Concentrations of Deltamethrin

The lethal concentrations were determined at all tested time points. The LC_50_ values of deltamethrin (topical exposure) were 86.28 ppm, 36.16 ppm, and 29.19 ppm at 12, 24, and 48 h, respectively. The LC_50_ values of deltamethrin (oral exposure) were 35.77 ppm, 32.53 ppm, and 30.78 ppm at 12, 24, and 48 h, respectively (Table 1). The sublethal concentrations of deltamethrin at 12, 24, and 48 h are presented in Table 2.

**Figure 6:**
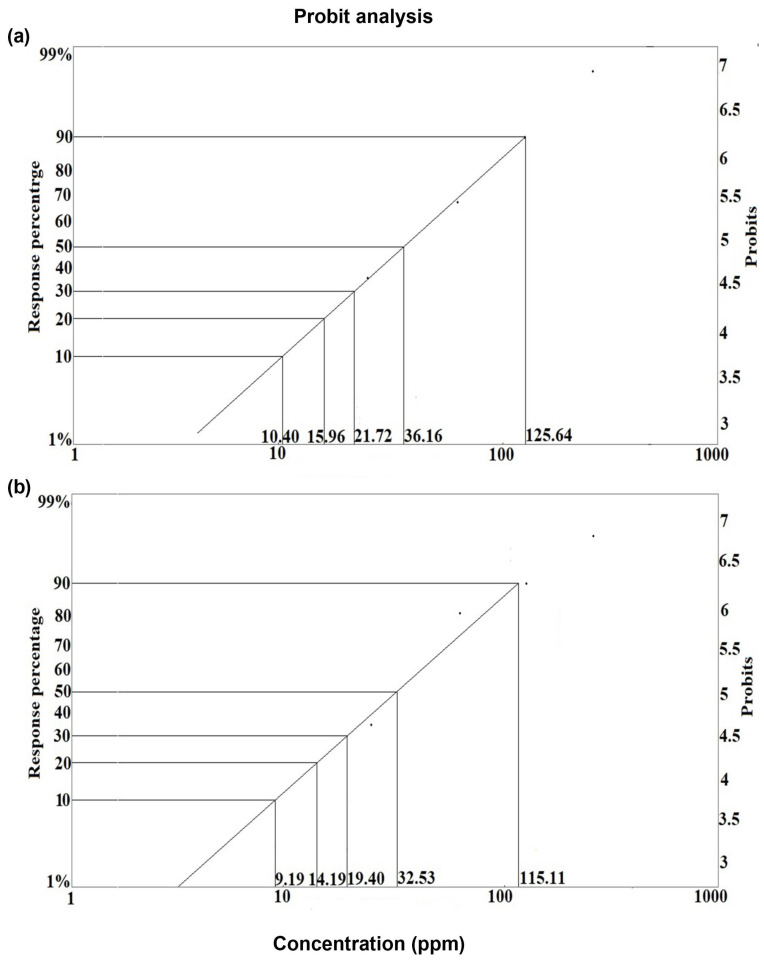
Probit analysis graph for lethal and sublethal concentrations of deltamethrin at 24 h posttreatment: (**a**) topical (**b**) oral exposure.

The graph of the probit analysis shows the values of the lethal and sublethal concentrations of deltamethrin at 24 h exposure time after topical (Figure 6a) and oral exposure (Figure 6b).

#### 3.1.5. Mortality of Bees: Topical vs. Oral Exposure

**Figure 7:**
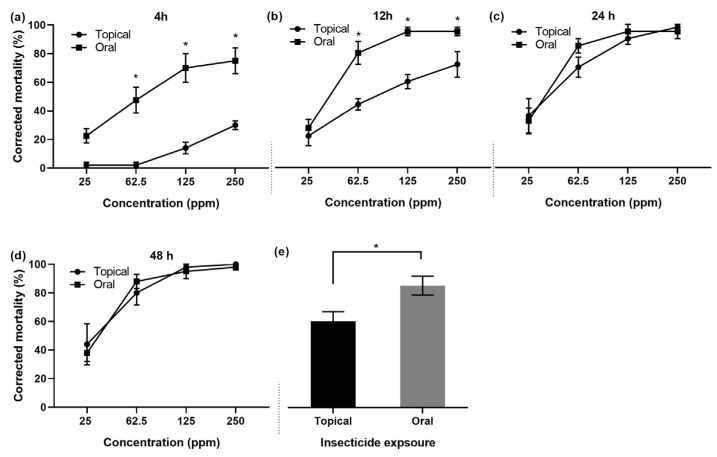
Mortality of *Apis mellifera jemenitica* after topical and oral exposure to deltamethrin (**a**) 4 h posttreatment, (**b**) 12 h posttreatment, (**c**) 24 h posttreatment, and (**d**) 48 h posttreatment; (**e**) aggregate mortality after topical and oral exposure. Asterisks represent a significant difference between the mortality of bees after topical and oral exposure at each single concentration attribute (*p <* 0.05, *t* test).

The mortality of bees after oral exposure to deltamethrin was significantly different at all tested dilutions compared to topical exposure at 4 h posttreatment (Figure 7a). Likewise, at 12 h posttreatment, oral exposure resulted in significantly higher bee mortality than topical exposure, except at 25 ppm (Figure 7b). Both exposure routes (topical and oral) were equally toxic to honey bees at 24 h (Figure 7c) and 48 h (Figure 7d) posttreatment. The aggregate mortality of *A. m. jemenitica*, irrespective of concentration and time, showed significantly higher mortality after oral exposure than topical exposure (Figure 7e).

### 3.2. Olfactory Behavior of Honey Bees

Sublethal concentrations (LC_10_, LC_20_, and LC_30_) of deltamethrin were applied as a single intake through two routes (topical and oral) to *A. m. jemenitica* honey bees. All tested concentrations of deltamethrin significantly affected the learning and memory of *A. m. jemenitica* when exposed through both topical and oral routes compared to the control treatment.

#### 3.2.1. Associative Learning and Topical Exposure to Deltamethrin

**Figure 8:**
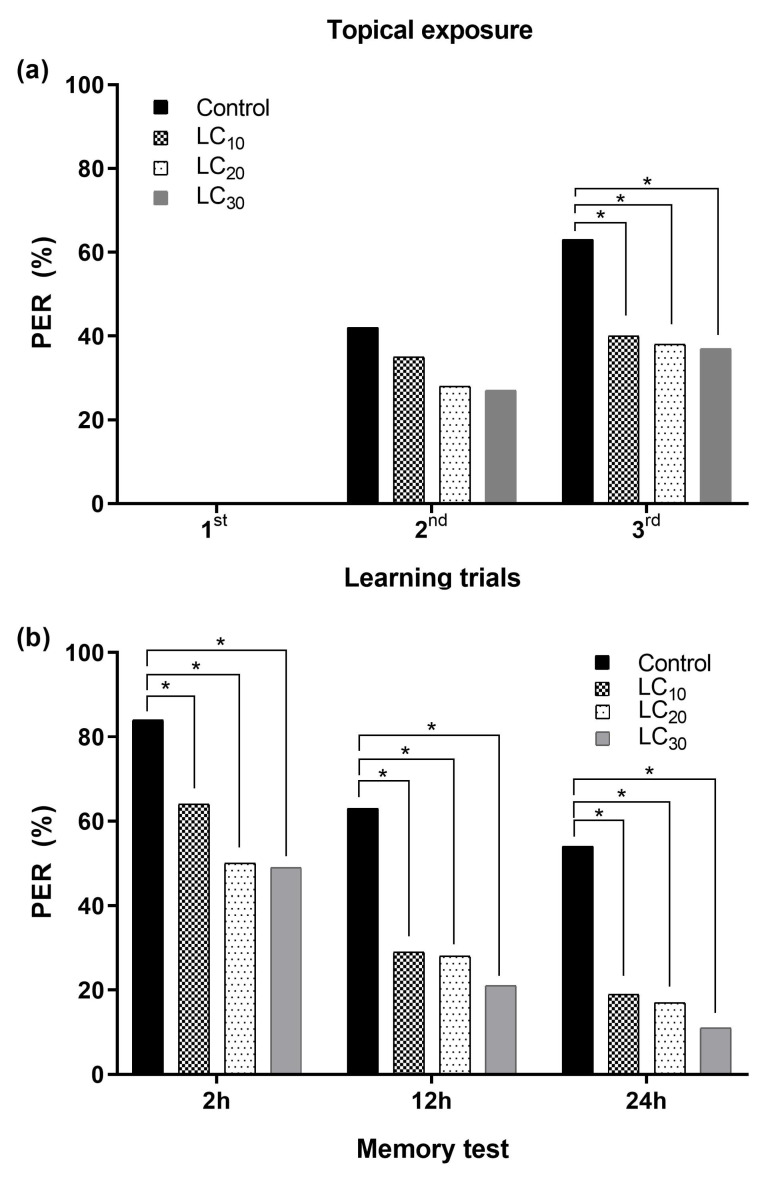
Proboscis extension response (PER) of *Apis mellifera jemenitica* after exposure to deltamethrin through the topical route: (**a**) learning trials, (**b**) memory formation test. The asterisk indicates significant differences between the control and deltamethrin-treated groups of bees (Fisher’s exact test/χ^2^ test; * *p* < 0.05).

Olfactory learning was significantly impaired with a sublethal concentration (LC_10_ = 10.40 ppm, LC_20_ = 15.95 ppm, and LC_30_ = 21.72 ppm) of deltamethrin topically applied on the thorax. The impairment in learning was concentration-dependent, and a high reduction in learning was observed with a higher sublethal concentration (LC_30_), followed by LC_20_ and LC_10_. PER of the treated bees gradually decreased along with an increase in the sublethal concentration during the learning trials (2nd–3rd) compared to the control. No bee elicited any learning response during the first learning trial in all tested group of bees. PER was significantly reduced during the 3rd learning trials with all three tested sublethal concentrations compared to the control group of bees. However, none of the three tested sublethal concentrations showed any significant differences among each other during all learning trials (Figure 8a). The sublethal concentrations also significantly reduced memory formation, with reduced PER in the 2 h, 12 h, and 24 h memory formation tests compared to the control group. Likewise, all three tested sublethal concentrations of deltamethrin were equally toxic to honey bees during all memory tests (Figure 8b).

#### 3.2.2. Associative Learning and Oral Exposure to Deltamethrin

**Figure 9:**
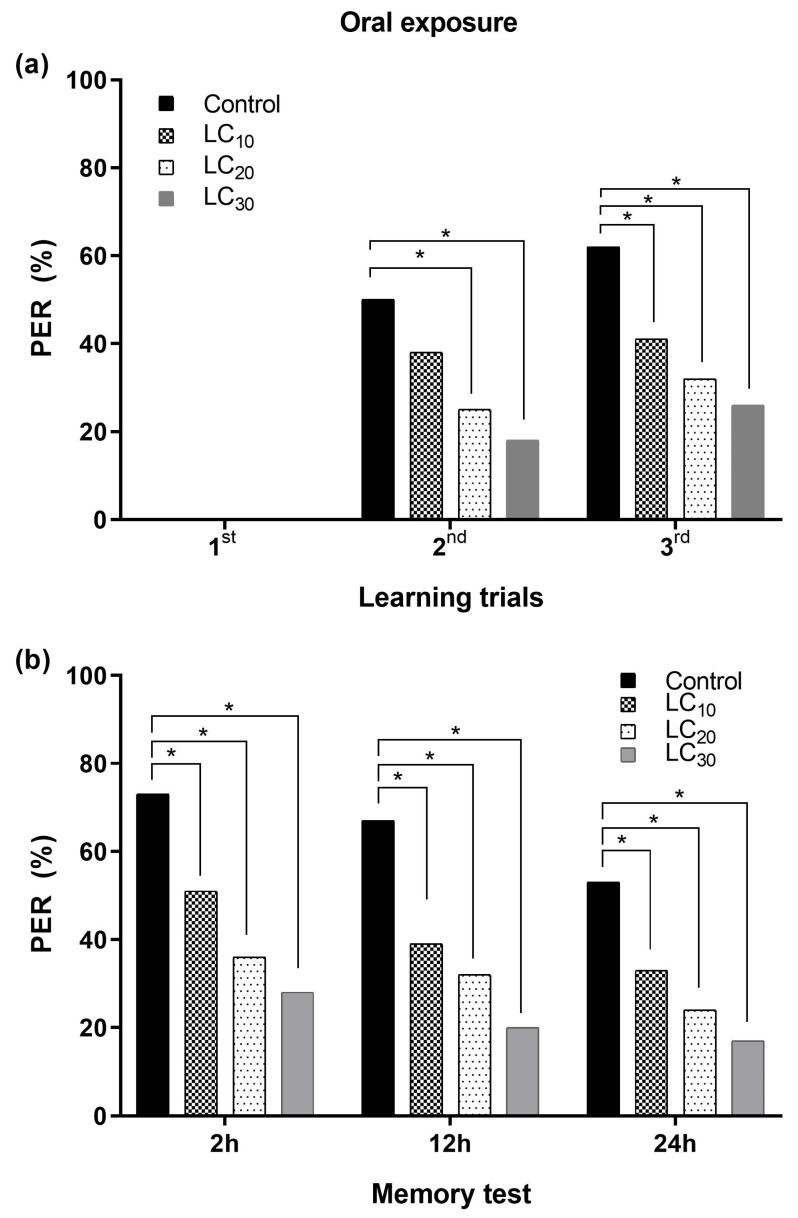
Proboscis extension response (PER) of *Apis mellifera jemenitica* after exposure to deltamethrin through the oral route: (**a**) learning trials, (**b**) memory formation test. The asterisk indicates significant differences between the control and deltamethrin-treated groups of bees (Fisher’s exact test/χ^2^ test; * *p* < 0.05).

Oral exposure to sublethal concentrations of deltamethrin (LC_10_ = 9.19 ppm, LC_20_ = 14.19 ppm, and LC_30_ = 19.40 ppm) significantly reduced learning and memory formation compared to the control. PER gradually decreased alongside an increase in sublethal concentration during learning trials. PER was significantly reduced during the 2nd–3rd learning trials in those dosed with the sublethal concentrations compared to the control group of bees. However, all three tested sublethal concentrations of deltamethrin were equally toxic to honey bees, with non-significant effects on the PER among them during all learning trials (Figure 9a). Memory formation with reduced PER compared to the control group was recorded for all tested sublethal concentrations during the memory formation tests at 2 h, 12 h, and 24 h. Likewise, in the learning trials, all three tested sublethal concentrations of deltamethrin were equally toxic to honey bees during all memory tests (Figure 9b).

## 4. Discussion

Honeybees play a crucial role in the pollination of plants and agricultural crops [78]. Bees are under serious threat due to extensive worldwide use of pesticides against crop, domestic, and horticultural insect pests [14,15]. Deltamethrin (pyrethroid insecticide) is widely used in mosquito control in Saudi Arabia [38,40]. Honey bees suffer from the devastating effects of insecticide toxicity. Considering the deleterious insecticidal effect, it is alarming that the native honey bee of Saudi Arabia, *A. m. jemenitica,* is at potential massive risk due to deltamethrin. We found that, when applied through two separate routes (topical and oral), deltamethrin significantly increased the mortality of bees and impaired olfactory learning and memory formation in *A. m. jemenitica*.

### 4.1. Topical Application and Mortality

Our study showed that topical application of deltamethrin was highly toxic to *A. m. jemenitica* at a realistic field dilution (250 ppm) and serial dilutions (125 ppm and 62.5 ppm) from 12–48 h. The mortality of *A. m. jemenitica* was significantly higher in treated bees (topical application of insecticide) than in the control group. Deltamethrin (Decis EC 2.5) was found to be highly toxic compared to methomyl, chlorpyrifos, and profenofos after 24–48 h of topical application to *A. mellifera* [79]. Deltamethrin was highly toxic (100% mortality of *A. mellifera*) just 1 h after being directly sprayed on the melon crop (*Cucumis melo* L.) [80]. In a previous study, 97.61% mortality of the Algerian honey bee *A. m. intermissa* was observed following a 50 ppm deltamethrin (Decis EC25) topical route dose after 24 h [81].

Direct (topical) or indirect exposure (contact through filter paper) to deltamethrin (Decis 2.8 EC) at the recommended field rate of 1.07 g/L (30 ppm) resulted in 100% mortality of both *Apis cerana* and *A. mellifera* at 24 h and 48 h posttreatment [82]. We also found that the field-recommended deltamethrin dilution as a topical application resulted in 98% and 100% mortality at 24 h and 48 h posttreatment, respectively. The high mortality of bees after topical application of insecticide could be due to its penetration ability through the insect cuticle, which can absorb up to 48% of the initially administered chemical substance on bees [83].

### 4.2. Oral Application and Mortality

Our research revealed that the oral ingestion of deltamethrin was highly toxic to *A. m. jemenitica*, with the highest mortality (95%, 95%, and 98%) at 12, 24, and 48 h posttreatment time, respectively, at the recommended field dilution and its serial dilutions. The mortality of *A. m. jemenitica* was significantly higher in treated bees (orally fed insecticide) than in the control group. A previous study found a lethal time (LT_50_) of 73.011 h with oral exposure to deltamethrin (2.50 ppm) [84]. Deltamethrin was moderately toxic to bees, with mortality rates ranging from 45.2 to 64.3% with an LT_50_ of 48.91 h [80]. The high mortality of bees after oral feeding with deltamethrin can be attributed to its toxic effects on their physiological functions and behavior [16].

Deltamethrin had a detrimental effect on the food consumption of *A. m. jemenitica*, and the increase in the ingested concentration corresponded to the gradually decreased consumption of contaminated food, and vice versa. Interestingly, low food consumption at a high concentration (250 ppm) was extremely toxic to the bees, with the highest mortality. Thus, the consumption of a lower quantity of food with a high concentration of deltamethrin was also lethal to honey bees. In agreement, deltamethrin (1 mL/day) significantly affected syrup consumption and foraging activity in *Apis mellifera* in an indoor cage experiment [85].

Additionally, our results agree with those of [86], who reported a reduction in sucrose intake when workers were administered sugar solutions containing 0.1–0.2 mg/kg (1 ppm) deltamethrin.

### 4.3. Comparison of Topical vs. Oral Exposure to Deltamethrin for Bee Mortality

The oral administration of deltamethrin was highly toxic to *A. m. jemenitica* compared to the topical route, especially at 4 h and 12 h posttreatment. However, both routes were equally toxic at 24 h and 48 h posttreatment regarding the mortality of bees. Shah et al. [68] also reported no significant difference in deltamethrin percent-corrected mortality between the exposure methods (oral and topical on the thorax) after 24 h and 48 h with lower concentrations of deltamethrin (1.11 µg/mL). However, oral exposure-induced mortality was lower than contact mortality for *A. mellifera* with higher concentrations (90 µg/mL) of deltamethrin. The topical application of deltamethrin also showed a lower impact on the mortality of *A. mellifera* when compared with its contact and residual method after 48 h [87]. In contrast, our study showed that at 4 and 12 h posttreatment, mortality was higher after the oral than the topical application of higher concentrations of deltamethrin.

### 4.4. Lethal Concentrations

The lethal concentration is a very useful indicator for measuring the lethality of particular pesticides and assessing sublethal exposure in honey bees [88]. We found that the LC_50_ of the commercial deltamethrin formulation (25EC) at the 24 h and 48 h posttreatment times after topical exposure were 36.16 ppm and 29.19 ppm, respectively, and after oral exposure were 32.53 ppm and 30.78 ppm, respectively, in *A. m. jemenitica*. Numerous studies have determined the lethal values of commercial formulations and active ingredients of deltamethrin in different experimental setups with various bee subspecies at different times and with different measuring scales. The topical application of deltamethrin (active ingredient) and its commercial formulation (deltaguard 2.0%) gave LD_50_ values of 0.013 µg/g and 0.055 µg/g, respectively [89,90]. Deltamethrin (Decis EC25), a commercial formulation, presented LC_50_ at 24 h and 48 h posttreatment after topical exposure (10.40 ppm and 7.38 ppm, respectively) and after oral exposure (20.92 ppm and 18.39 ppm, respectively) in *A. m. intermissa* [81]. Acute toxicity of topically applied deltamethrin (98% a.i.) against *A. mellifera* showed lethal values of 50.65 ng/bee at 48 h posttreatment [91].

Deltamethrin (Decis 25 EC) gave an LC_50_ of 112.2 μg a.i./bee (topical application) and an LD_50_ of 0.850 μg a.i./bee (oral application) at 24 h posttreatment on *A. mellifera* (Del Sarto et al. [63]. Oral administration of deltamethrin Decis (25 g of active ingredients) for 5 h in the Africanized honeybee *Apis mellifera* revealed an LD_50_ (18.4 ng a.i./bee) at 24 posttreatment [92]. The contact LD_50_ of deltamethrin in honey bees was 0.051 g/bee under laboratory conditions [93]. In the present study, the LC_50_ of deltamethrin (25EC) was 30.78 ppm at 48 h after oral exposure. A lower formulation (2.5EC) of deltamethrin showed a high LC_50_ of 60.8 mg/L (ppm) at 48 h after oral administration to *Apis mellifera ligustica* [24].

The differences in lethal values might be due to factors such as honey bee subspecies, bee age, commercial insecticide formulations, temperature, season of treatment, and experimental conditions [94].

### 4.5. Associative Learning of Honey Bees

The bees exposed to sublethal concentrations through topical or oral routes showed drastic changes in their learning and memory formation, with a reduction in their proboscis extension response (PER) as compared to the untreated bees. Thus, irrespective of the exposure route in the field, deltamethrin is toxic and negatively affects foraging, learning, and memory formation in honey bees.

#### 4.5.1. Topical Route and Learning and Memory

In our results, topical application of sublethal concentrations of deltamethrin (LC_10_, LC_20_, and LC_30_) significantly affected the learning trials and memory formation tests of *A. m. jemenitica*. The treated bees showed lower responses (PER) during learning trials (2nd–3rd) and memory formation (2 h, 12 h, and 24 h) than untreated bees. Likewise, sublethal concentrations of deltamethrin mixed with a 50% sucrose solution showed a negative effect on learning and memory [95]. *A. mellifera* treated with sublethal doses of permethrin (pyrethroid) mixed with 25% sucrose solution exhibited a lower learning response (up to 3 days) than those left untreated [96]. Pyrethroid exposure to *Apis mellifera* causes significant colony mortality and a reduction in learning and memory [97]. Exposure of *Apis mellifera* foragers to lambda-cyhalothrin (pyrethroid) at the rates of ½ LC_50_ and ¼ LC_50_ significantly impaired their PER compared to the control group [98].

#### 4.5.2. Oral Route and Learning and Memory

We found that deltamethrin was highly toxic at three tested sublethal concentrations during learning trials and memory formation tests, which led to the conclusion that oral application of deltamethrin has a significant effect on the cognitive ability of *A. m. jemenitica*. In contrast, learning remained unchanged in *Apis mellifera* upon oral intake of deltamethrin (100 ng/bee) 24 h before learning or during learning, but memory formation was significantly different in both cases [99]. However, our results depicted the significant differences in learning and memory formation of *A. m. jemenitica* when insecticide was ingested orally 1 h before learning. Deltamethrin interferes with the nervous system, causing disorders in dancing communication and foraging activity, and its sublethal dose (235 µg/kg) impairs learning and memory [45]. A deltamethrin (960 µg/L) dose mixed with 50% sucrose significantly impaired olfactory learning performance in bees during conditioning trials [23]. Acute oral toxicity of deltamethrin resulted in the highest toxicity and disturbance of honeybee function and behavior after the permeation of toxins at sublethal doses [100].

Thus, sublethal concentrations of deltamethrin appear to be significantly harmful to honey bees by decreasing their cognitive abilities (learning and memory). As per numerous studies, deltamethrin is also known to affect other physiological and behavioral processes of honey bees, such as reducing queen fecundity, prolonging larval development, reducing lifespan, impairing honeybee dance and foraging ability, disturbing thermoregulation, and causing hypothermia in honey bees [24,44].

## 5. Conclusions

Conclusively, sublethal concentrations of deltamethrin cause severe negative effects on learning, and bees were unable to memorize the learned task. Deltamethrin is highly toxic, causing high mortality in native bees (*A. m. jemenitica*) of Saudi Arabia through both topical and oral routes. The mortality of bees was directly associated with the concentration of insecticide: the higher the concentration, the higher the bees’ mortality, and vice versa. The effect of oral ingestion is faster and more visible at 4 h than topical application. The bees’ preference for contaminated food gradually decreased with increasing concentration of deltamethrin. Thus, the reduction percentage of food intake is directly related to the administered oral concentration of deltamethrin. Oral administration of a higher concentration of deltamethrin resulted in decreased bee food consumption but high toxicity, indicating that a small amount of contaminated food is enough to cause bee mortality. The sublethal concentrations of deltamethrin, when fed orally or topically, considerably impair olfactory associative learning and memory formation. There is a need to increase awareness among people regarding the toxicity of insecticides against honey bees, and a need to strictly monitor standard precautionary measures with minimum passive use of insecticides.

## Figures and Tables

**Table 1:** Lethal concentrations of deltamethrin at 12, 24, and 48 h after topical and oral application.

Exposure Mode	Time (h)	LC_50_ (ppm)	LC_90_ (ppm)
Topical	12	86.28	759.46
	24	36.16	125.65
	48	29.19	79.62
Oral	12	35.77	193.37
	24	32.53	115.11
	48	30.78	83.24

**Table 2:** Sublethal concentrations of deltamethrin at 12, 24, and 48 h after topical and oral application.

Time (h)	Sublethal Concentration (ppm)					
	Topical Exposure			Oral Exposure		
	LC_10_	LC_20_	LC_30_	LC_10_	LC_20_	LC_30_
12	9.80	20.68	35.43	6.61	11.81	17.93
24	10.40	15.95	21.72	9.19	14.19	19.40
48	10.70	15.10	19.36	11.83	16.02	20.49

## Data Availability

Data is contained within the article.
